# *GSTM1* null genotype underpins recurrence of *NF2* meningiomas

**DOI:** 10.3389/fonc.2024.1506708

**Published:** 2024-12-12

**Authors:** Anthony C. Johnson, Erdyni N. Tsitsikov, Khanh P. Phan, Jeffrey A. Zuccato, Andrew M. Bauer, Christopher S. Graffeo, Sanaa Hameed, Tressie M. Stephens, Yufeng Liu, Gavin P. Dunn, Alla V. Tsytsykova, Pamela S. Jones, Ian F. Dunn

**Affiliations:** ^1^ Department of Neurosurgery, University of Oklahoma Health Sciences Center, Oklahoma City, OK, United States; ^2^ Department of Physiology, University of Oklahoma Health Sciences Center, Oklahoma City, OK, United States; ^3^ Department of Neurosurgery, Massachusetts General Hospital, Harvard Medical School, Boston, MA, United States

**Keywords:** meningioma, recurrent, CNS tumors, transcriptome profiling, gene expression, *NF2*, *GSTM1*

## Abstract

**Introduction:**

Meningiomas are the most common primary central nervous system (CNS) tumor in adults, comprising one-third of all primary adult CNS tumors. Although several recent publications have identified molecular alterations in meningioma including characteristic mutations, copy number alterations, and gene expression signatures, our understanding of the drivers of meningioma recurrence is limited.

**Objective:**

To identify gene expression signatures of 1p^-^22q^-^NF2^-^ meningioma recurrence, with concurrent biallelic inactivation of *NF2* and loss of chr1p that are heterogenous but enriched for recurrent meningiomas.

**Methods:**

Transcriptomic alterations present in recurrent versus primary 1p^-^22q^-^NF2^-^ meningiomas were identified using RNA sequencing (RNA-seq) data in a clinically annotated cohort.

**Results:**

Recurrent 1p^-^22q^-^NF2^-^ meningiomas were enriched for a newly identified *GSTM1* null genotype compared to primary meningiomas that showed variable *GSTM1* expression and independent external validation was performed.

**Conclusions:**

The *GSTM1* null genotype is a novel biomarker of 1p^-^22q^-^NF2^-^ meningioma recurrence that resolves heterogeneity in existing meningioma subtypes and may be used to guide future clinical management decisions on extent of treatment to improve patient outcomes.

## 1 Introduction

Meningiomas are tumors that originate in the meninges and are the most common intracranial tumor type in adults, representing 39% of all primary adult central nervous system (CNS) tumors (1). The World Health Organization (WHO) classifies meningiomas into 15 subtypes and grades 1-3 based on histopathological features and specific molecular alterations (2). Alterations in *NF2, moesin-ezrin-radixin like (MERLIN) tumor suppressor*, are the most common genetic abnormality in meningioma, with up to 60% of sporadic meningiomas harboring them (3, 4). *NF2* is mapped to chromosome 22 (chr22) and encodes merlin, an intracellular scaffold protein and tumor suppressor (5). Biallelic gene inactivation of *NF2* resulting from chr22 monosomy and concurrent mutations in the remaining *NF2* allele are characteristic alterations in multiple central and peripheral nervous system tumors in addition to meningioma including schwannomas and ependymomas (6). Allelic loss of chromosome 1p (chr1p) is the second most commonly observed chromosomal abnormality in meningiomas after deletion of chr22 (7, 8). Additional frequent genomic alterations in meningioma include mutations in *TNF receptor associated factor 7* (*TRAF7*), *KLF transcription factor 4* (*KLF4*), and *phosphatidylInositol-4,5-bisphosphate 3-kinase catalytic subunit alpha* (*PIK3CA*) (9, 10).

Recent advances in high throughput molecular profiling, including epigenetic, cytogenetic, and gene expression analyses, combined with computational modeling approaches have enabled enhanced classification of meningiomas into molecular subtypes that more accurately represent their clinical behavior than traditional histopathological evaluation (11, 12). A prominent molecular meningioma classification separates tumors into 3 subtypes: Type A meningiomas with *TRAF7*, *KLF4*, and/or *AKT serine/threonine kinase 1* (*AKT1)* missense mutations and without significant chromosomal copy number alteration; type B meningiomas primarily distinguished by *NF2* loss; and type C meningiomas with biallelic *NF2* inactivation plus loss of chromosome 1p (13, 14). Different chr1p regions have been reported to be associated with meningioma development including 1p36, 1p34-1p32, 1p22, and 1p21.1-1p13 (15–18),, but the most frequent loss is in 1p34.1 (19, 20). Chromosome 1p loss leads to reduced expression of several genes including *patched 2* (*PTCH2)* (21), *AT-rich interaction domain 1A* (*ARID1A)* (22, 23), *alkaline phosphatase, biomineralization associated* (*ALPL)* (24, 25), and *cyclin dependent kinase inhibitor 2C* (*CDKN2C)* (26).Type A and B meningiomas tend to follow a more benign course and type C are enriched for recurrent meningiomas. These subtypes better predicted tumor recurrence than WHO grading, the current clinical standard for predicting tumor recurrence. However, type C meningiomas with a higher recurrence risk remain a heterogenous group, with one-half recurring within 5 years and the other half exhibiting up to 5 years of recurrence free survival (13, 14). Another study divided meningiomas into four stable molecular groups (MG1-4) with type C meningiomas separated into two most clinically aggressive groups: hypermetabolic (MG3) and proliferative (MG4) (12, 13). The most frequent chromosomal abnormality in both MG3 and MG4 was the loss of both chr22q and chr1p (27). Novel somatic driver events identified in these meningiomas were chromatin remodeling and mutations in epigenetic regulators *lysine demethylase 6A* (*KDM6A*), *chromodomain helicase DNA binding protein 2* (*CHD2*), and *tumor suppressor phosphatase and tensin homolog* (*PTEN*).

Finally, another subclassification of type C meningiomas based on transcriptomic data identifies A1 and A2 tumors distinguished by a 34-gene expression risk signature, with the latter group having a higher proportion of recurrent tumors (27). For this work, 13 bulk RNA-seq datasets were combined to create a dimension-reduced reference landscape of 1,298 meningiomas (28). The resulting reference map exhibited multiple clusters of tumors, indicating multiple RNA-seq-based meningioma subtypes, some of which were associated with distinct time to recurrence. The most striking differences of time to recurrence was seen between subclusters A1 and A2 within cluster A. While both A1 and A2 harbored meningiomas with biallelic inactivation of *NF2* and loss of chr1p, subcluster A2 contained a much larger population of patients with poor outcomes compared to subcluster A1 (28). Interestingly, while A1 and A2 can be distinguished based on 34-gene expression risk signature, no single specific causative genetic alteration was identified (29).

Here, we directly compare transcriptional profiles of recurrent and primary 1p^-^22q^-^NF2^-^ meningiomas, building on recent molecular advances in meningioma classification by focusing on this subtype enriched for recurrent tumors. The most differentially expressed gene (DEG) was *glutathione S-transferase mu 1 (GSTM1)*, encoding a member of the mu class of cytosolic glutathione S-transferase (GST) family of metabolic isozymes responsible for detoxification of a broad range of substances including environmental toxins, drugs, and carcinogens. GSTs are divided into three major protein families with multiple subclasses in each family (30). Intensive study of *GSTM1* has found frequent genetic polymorphisms, including complete biallelic deletion of the *GSTM1* loci (31, 32).

Our results demonstrated that five out of twelve primary meningiomas had no expression of *GSTM1*, suggesting that these patients’ meningioma might reoccur in the future. The comparison of *GSTM1*-negative (*GSTM1^-^*) and *GSTM1*-positive (*GSTM1^+^*) transcriptomes revealed that *GSTM1^-^* meningiomas were characterized by higher expression of genes specific for positive regulation of cell motility, locomotion and angiogenesis, while transcriptomes of *GSTM1^+^* tumors were enriched for transmembrane transport and system process genes. Taken together, our results present evidence that *GSTM1* expression was completely absent in all recurrent meningiomas due to the *GSTM1* null genotype resulting from inherited gene deletion and/or somatic deletion of chr1p, where *GSTM1* is mapped.

Overall, existing work has largely focused on classifying meningiomas based on molecular alterations within primary tumors to predict the clinical presentation and course of the subtypes. This has transformed our understanding of the spectrum of meningioma clinical behavior but has not identified definitive biomarkers of meningioma recurrence, which are required in order to enable personalized medicine approaches to escalate treatment in aggressive meningiomas with the aim to delay recurrence and improve patient outcomes. This study identifies *GSTM1* as a new biomarker of meningioma recurrence that builds on existing molecular subtypes to improve our ability to predict and manage recurrent meningiomas.

## 2 Materials and methods

### 2.1 Patients and sample collection

All procedures were approved by the institutional review board (IRB) of the University of Oklahoma Health Sciences Center (OUHSC) (IRB protocol number 10195). Sixteen samples from 16 patients (one sample/patient) diagnosed with meningioma at University of Oklahoma Medical Center were included in the study. All patients provided written informed consent for participation in the study. Clinical information including patient demographics, clinical course, and neuropathology was collected by retrospective chart review.

### 2.2 Histopathologic grading and genetic profiling

Following routine pathology processing, resected meningiomas were assigned a histopathologic grade according to the revised 4^th^ edition of the WHO Classification of Tumors of the CNS (2). Ki-67 immunostaining was performed on at least one block in all cases. All samples were analyzed, graded, and independently confirmed by two staff neuropathologists. For genetic profiling of each tumor, specimens were sent to the Mayo Clinic Laboratories. Somatic mutations and gene rearrangements were examined by the NONCP panel. Copy number imbalances and loss of heterozygosity were estimated by a CMART panel. For RNA extraction, resected tissues were immediately submerged in RNAlater^®^ Solution, kept at room temperature for 24 hours, and stored frozen long term (Fisher Scientific, AM7023).

### 2.3 RNA-seq and differential expression analysis

*GST* gene mRNA levels were calculated relative to *glyceraldehyde-3-phosphate dehydrogenase (GAPDH)* mRNA levels measured by RNA-seq for *GSTM* genes and multiplex RT-qPCR for *GSTT* genes. Total RNA was extracted from tumors saved in RNAlater^®^ Solution (Fisher Scientific, AM7023) with the RNeasy Plus mini kit (QIAGEN, 74136) with QIAshredder (QIAGEN, 79656). Preparation of cDNA libraries and sequencing was conducted by Novogene Co., LTD (Beijing, China). Significant DEGs were defined as those that had both an absolute log2FoldChange ≥ 1 as well as a false discovery rate adjusted p-value ≤ 0.05 for each comparison independently. Gene expression levels are expressed as Fragments Per Kilobase of transcript per Million mapped reads (FPKM). Pearson correlation analysis of the FPKM values for *GSTM1 vs*. *GSTM2* expression was performed in GraphPad (v10.4) with p <0.05 considered statistically significant.

### 2.4 Quantitative PCR

Total tumor RNA was used to measure gene mRNA levels by real-time qPCR. Reverse transcription and cDNA amplification were performed in one tube using qScript™ XLT One-Step RT-qPCR ToughMix^®^, Low ROX™ (VWR Quanta Biosciences™, 95134) on an Applied Biosystems 7500 Fast Real-Time PCR System (Fisher Scientific). Sample reactions were run in 3-6 replicates. Each mRNA analysis was run in a DuPlex PCR reaction with *GAPDH* as an internal control. Standard curves for each gene were run to verify the linear range of amplification. Input RNA was kept under 200 ng per reaction to stay within the linear range for *GAPDH* levels. Gene expression levels for all genes of interest were determined by comparative ΔCT experiment runs, analyzed using the 7500 Software v2.3 and calculated as Relative Quantity (RQ). Shown expression values represent RQ multiplied by 1000. Pearson correlation analysis of RQ values for *GSTM1 vs*. *GSTM4* expression was performed in GraphPad (v10.4) with p <0.05 considered statistically significant. Primers and Probes sequences used:

*GSTT1*-Fwd: TCCTTACTGGTCCTCACATCTC
*GSTT1*-Rev: GGCCTTCGAAGACTTGGC
*GSTT1* Probe: ATGCATCAGCTCCGTGATGGCTAC (FAM – BHQ1)
*GSTT4*-Fwd: TCATCACCGGGAACCAAATC
*GSTT4*-Rev: GGAGCTGTTGAGGAAGACATTAT
*GSTT4* Probe: TGGTGGAGATGATGCAGCCCAT (FAM – BHQ1)
*GAPDH*-Fwd: GGTGTGAACCATGAGAAGTATGA
*GAPDH*-Rev: GAGTCCTTCCACGATACCAAAG
*GAPDH* Probe: AGATCATCAGCAATGCCTCCTGCA (VIC-TAMRA)

### 2.5 Determination of *GST(x)* genes copy number by real-time PCR

Genomic DNA was extracted from tumor tissues with the DNeasy Blood and Tissue kit (Qiagen, 69506). Exon/intron junction or intron regions from genes of interest were detected by a duplex qPCR (FAM/VIC TaqMan^®^ assay) for simultaneous detection of each gene of interest paired with *apolipoprotein B (APOB)* as a 2-copy standard reference gene. Standard curves were obtained by serial dilutions of a plasmid containing amplicon sequences of all genes of interest (*GSTT1, GSTT4, GSTM1, GSTM2*) and *APOB* sequence. The number of gene copies per cell (i.e. diploid genome) was determined by multiplying the ratio *GST(x)/APOB* by two. Primers and Probes sequences used:

*GSTT1*-Fwd: TCCTTACTGGTCCTCACATCTC
*GSTT1*-Rev: GGCCTTCGAAGACTTGGC
*GSTT1* Probe: ATGCATCAGCTCCGTGATGGCTAC (FAM-BHQ1)
*GSTT4*-Fwd: TCATCACCGGGAACCAAATC
*GSTT4*-Rev: GGAGCTGTTGAGGAAGACATTAT
*GSTT4* Probe: TGGTGGAGATGATGCAGCCCAT (FAM-BHQ1)
*GSTM1*-Fwd: CTGGGCATGATCTGCTACAA
*GSTM1*-Rev: TGTGCAGGAATGCAAGAGT
*GSTM1* Probe: AGTGAGCTGCATCTGACAGAGTTTGG (FAM-BHQ1)
*GSTM2*-Fwd: CAAACTCTGCTATGACCCAGAT
*GSTM2*-Rev: AAGACCAAGAACTCACCAGAAG
*GSTM2* Probe: CCTTTCCCTGCAGAGTTTGTGTCCA (FAM-BHQ1)
*H-ApoB* Fwd: TGAAGGTGGAGGACATTCCTCTA
*H-ApoB* Rev: CTGGAATTGCGATTTCTGGTAA
*H-ApoB* Probe: CGAGAATCACCCTGCCAGACTTCCGT (VIC-TAMRA)

## 3 Results

### 3.1 Clinical cohort characteristics

We built a cohort of sixteen patients with confirmed 1p^-^22q^-^NF2^-^ meningioma samples for our analysis. Clinical and pathological features are outlined in (**Table 1**). The primary meningioma group consisted of twelve patients, seven females (PF1 to PF7) and five males (PM1 to PM5). The recurrent meningioma patients included 1 female (RF1) and 3 males (RM1, RM2, and RM4). RM3 was analyzed and excluded from further analysis because it did not pass RNA quality control (RNA Integrity Number (RIN) <3). The median RIN for the included samples was 9.2 (**Table 1**). The median age at the time of surgery for recurrent tumors was 58 and the median time to recurrence after the initial surgery was 12 years.

**Table 1:** Clinical and pathological cohort characteristics.

Tumor Group	Patient ID	Sample ID	Patient Sex	Patient Age	CNS WHO grade	Histological Type	Ki-67 labeling index	RIN	Mutated Genes	Copy Number	
										Chr 22q	Chr 1p
Recurrent	RF1	M-105	F	69	1	Choroid	1	9.1	*NF2, SMARCAL1*	Loss of chr 22	Loss of 1p36.33p21.3 (including ARID1A)
	RM1	M-020	M	24	1	Atypical	3	8.2	*NF2, FGFR3*	Loss of chr 22	Loss of 1p36.33p11.2, partial loss of 1q21.1q44
	RM2	M-077	M	76	3	Atypical	3	9.7	*NF2, TERT, PTCH1, ARID1A, LRP1B, TET1, YAP1*	Loss 22q11.1q13.2	Loss of 1p36.33p11.2 (including ARID1A)
	RM4	M-112	M	47	2	Choroid	3	9.2	*NF2*	Loss of ch r22	Loss of 1p36.33p13.3 (including ARID1A)
Primary	PF1	M-015	F	57	1	Meningiothelial	2	9.8	*NF2, COL6A3*	Loss of chr 22	Multiple gains and losses on chr1 (including loss of ARID1A)
	PF2	M-064	F	61	1	Fibroblastic	<1	9.7	*NF2, CHEK2, SMARCB1, ARID2, MSH3*	Loss of ch 22	Loss of 1p36.33p22.1 (including ARID1A)
	PF3	M-071	F	63	2	Meningiothelial	<10	5.1	*NF2, CHEK2, NF1, KMT2B, KMT2D, PTCH2*	Loss of ch 22	Loss of 1p36.33p12
	PF4	M-073	F	46	1	Fibrous	2	9.8	*NF2, GL12, KDM5C, PARP1*	Loss of most of ch 22	Loss of most of 1p, loss of 8p23.3q12.3
	PF5	M-089	F	86	2	Meningiothelial	10	9.2	*NF2, EGFR, POLE*	Loss of chr 22	Loss of 1p36.33p31.1 (including ARID1A)
	PF6	M-097	F	64	1	Meningiothelial	5	8.0	*NF2*	Loss of chr 22	Loss of 1p36.32p32.2 (including ARID1A)
	PF7	M-122	F	60	1	Transitional	1	5.8	*NF2, FUBP1, VHL*	Loss of 22q12.1q13.33	Loss of 1p36.33p12
	PM1	M-037	M	66	1	Meningiothelial	3	9.8	*NF2, KLF4, KMT2B*	Loss of chr 22	Loss of 1p36.33p21.1 (including ARID1A)
	PM2	M-055	M	79	2	Atypical	7	6.4	*NF2, TET2*	Loss of chr 22	Loss of 1p36.33p31.1 (including ARID1A), loss of 1q23.2q44
	PM3	M-074	M	80	2	Atypical	10	9.7	*NF2, BCOR, MLH1*	Loss of chr 22	Loss of 1p36.33p21.1 (including ARID1A)
	PM4	M-092	M	83	1	Transitional	<1	9.1	*NF2*	Loss of chr 22	Loss of 1p36.33p31.3
	PM5	M-098	M	41	2	Atypical	12	8.5	*NF2, TET1*	Loss of 22q11.21q13.33	Loss of 1p36.33p13.1 (including ARID1A)

### 3.2 Transcriptomic signatures in recurrent versus primary meningiomas

**Figure 1:**
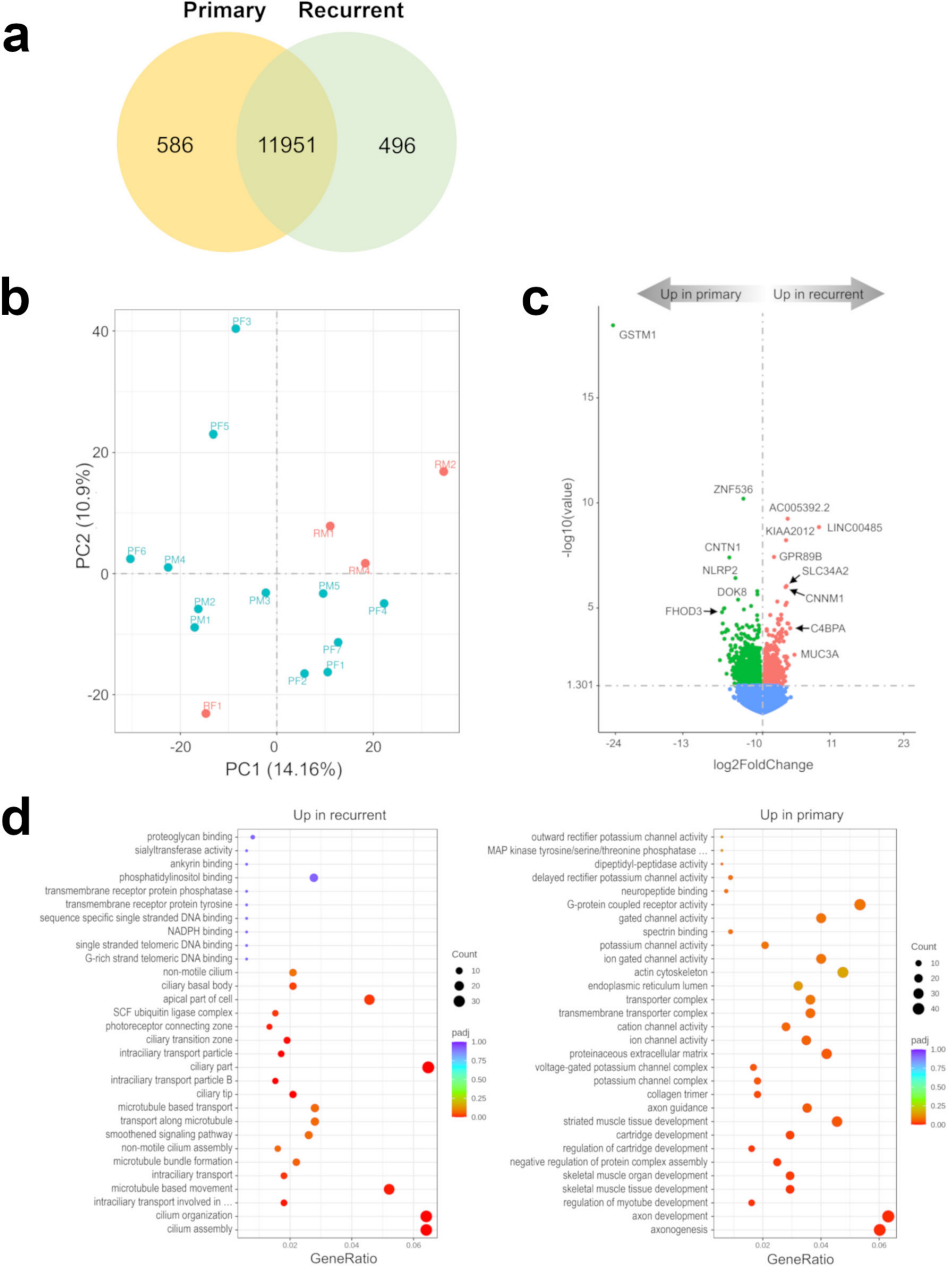
Recurrent 1p-22q-NF2- meningiomas have distinct transcriptomic markers. **(A)** Venn diagram showing the overlap of DEGs in recurrent and primary meningiomas by RNA-seq analysis. Common and tumor type-specific genes are depicted by numbers inside the corresponding circles. **(B)** PCA plot of recurrent (red dots) and primary (aquamarine dots) meningiomas using RNA-seq data. **(C)** Volcano plot displaying significant DEGs in recurrent *vs*. primary meningiomas. **(D)** Gene-set enrichment analyses of recurrent *vs*. primary meningiomas showing pathways that are upregulated and downregulated in recurrent meningioma. Bubbles represent pathways and gene ratios are percentages of significant genes of all genes in a pathway.

To evaluate differences in the transcriptome of recurrent versus primary 1p^-^22q^-^NF2^-^ meningiomas, we performed a differential comparison using RNA-seq data. We found that a vast majority of genes (11,951) were expressed in both primary and recurrent tumors, while relatively small number of genes (<8.3%) were expressed exclusively in primary (586 genes) or recurrent (496 genes) meningiomas (**Figure 1A**; **Supplementary File 1**). Nevertheless, the cohort showed a trend towards separation by recurrence status using this set of differentially expressed genes (DEGs) in a principal component analysis (PCA) plot (**Figure 1B**) and with hierarchical clustering (**Supplementary Figure S1**) and high Pearson correlation coefficient (**Supplementary Figure S2**).The most significant DEG was *GSTM1* (**Figure 1C**) that was downregulated in recurrent tumors. These results suggest that *GSTM1* under expression may be a biomarker of recurrence in 1p^-^22q^-^NF2^-^ meningiomas, which are difficult to prognosticate currently. Other significant DEGs included z*inc finger protein 536* (*ZNF536)* under expression (encoding a highly conserved transcription factor shown to negatively regulate neuronal differentiation) (33), *AC005392.2* and *LINC00485* long intergenic non-protein coding RNA overexpression, and *KIAA2012* overexpression in recurrent meningiomas (encoding an uncharacterized protein of unknown function highly expressed in excitatory neurons, choroid plexus, and other ciliated cell types) (proteinatlas.org). Gene set enrichment analyses across three gene set databases (GO, KEGG, and Reactome) overall showed overexpression of cell structure and signaling pathways and under expression of developmental pathways in recurrent meningiomas (**Figure 1D**; **Supplementary Figure S3**). Interestingly, KEGG database analysis failed to identify any significantly enriched pathways in recurrent meningiomas while primary tumors were significantly enriched in “cAMP signaling pathway”, “Notch signaling pathway” and “protein digestion and absorption,” underscoring the value of a multi-database pathway analysis.

### 3.3 Transcriptomic signatures in *GSTM1^-^* versus *GSTM1^+^* meningiomas

**Figure 2:**
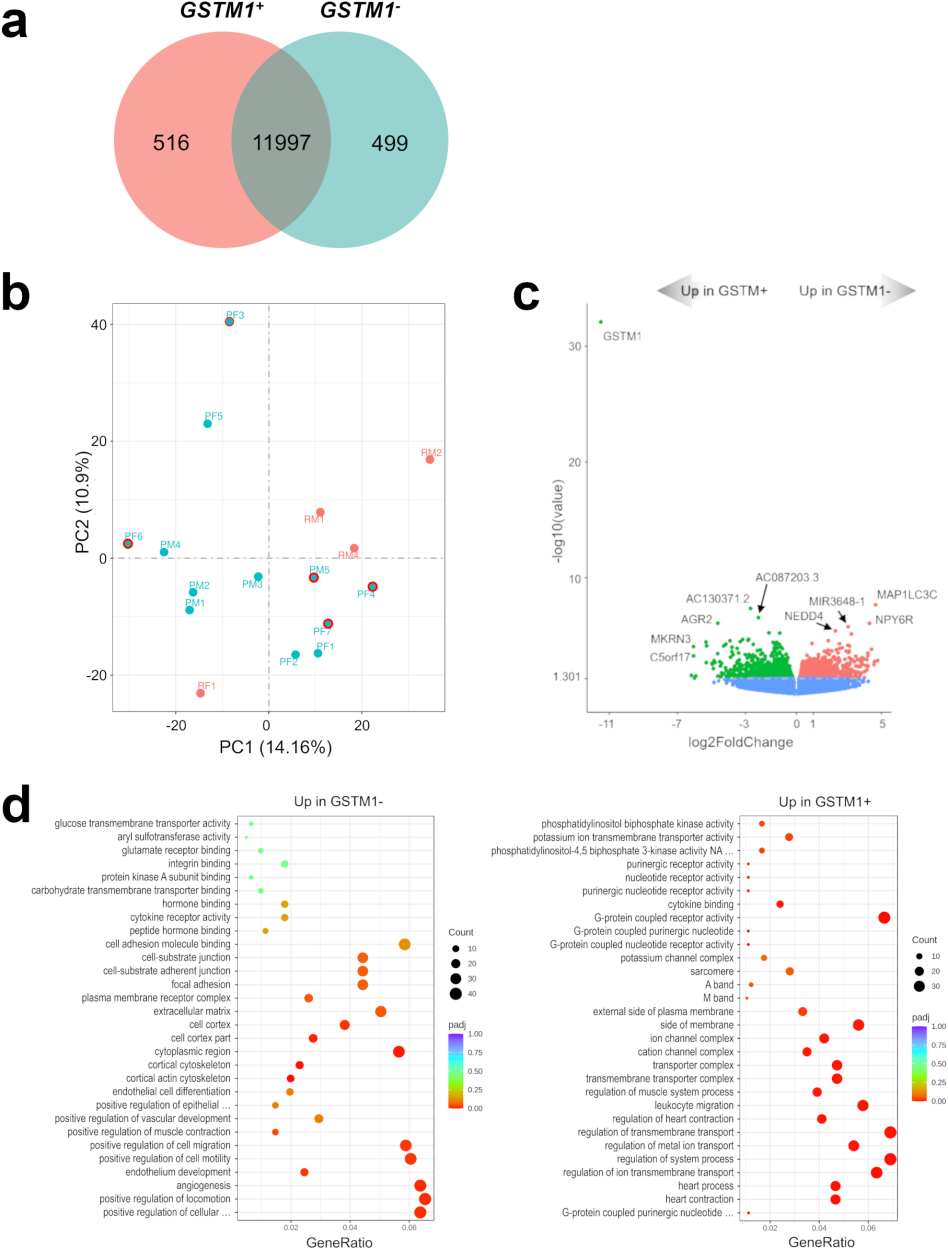
Characterization of the *GSTM1^-^* 1p^-^22q^-^NF2^-^ meningioma transcriptome. **(A)** Venn diagram showing the overlap of DEGs in *GSTM1^+^* and *GSTM1^-^* meningiomas by RNA-seq analysis. Common and tumor type-specific genes are depicted by numbers inside the corresponding circles. **(B)** PCA plot of RNA-seq analysis in *GSTM1^+^* and *GSTM1^-^* meningiomas. As in **Figure 1B**, all recurrent meningiomas are indicated by red dots and all primary tumors are indicated by aquamarine dots. In addition, primary tumors without *GSTM1* expression have a red outline. **(C)** Volcano plot displaying significant overexpressed and under expressed DEGs in *GSTM1^-^* meningiomas. **(D)** Gene-set enrichment analysis (GO) showing pathways upregulated and downregulated in *GSTM1^-^* meningiomas. Bubbles represent pathways and gene ratios are percentages of significant genes of all genes in a pathway.

**Figure 3:**
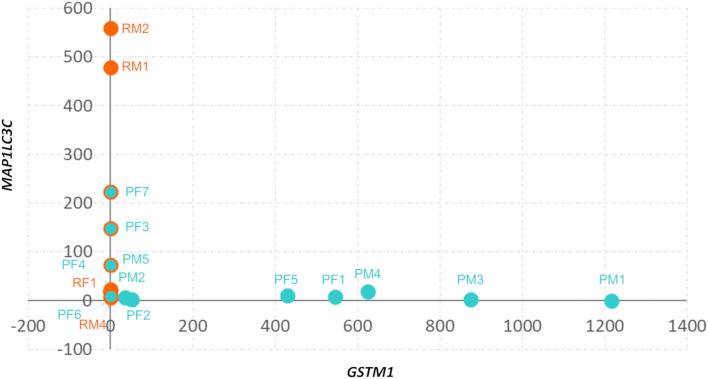
Divergent expression of *GSTM1* and *MAPLC3C* in 1p^-^22q^-^NF2^-^ meningiomas. Scatter plot of *GSTM1* and *MAP1LC3C* expression in 1p^-^22q^-^NF2^-^ meningiomas. Recurrent meningiomas are indicated by red dots, primary tumors are indicated by aquamarine dots, and primary tumors without *GSTM1* expression have a red outline.

All recurrent tumors demonstrated the absence of *GSTM1* expression and seven of 12 primary meningiomas showed *GSTM1* expression (**Supplementary File 2**). Next, we compared mRNA expression profiles of *GSTM1^-^* and *GSTM1^+^* subgroups to characterize differences between meningiomas with and without our marker of recurrence. There were 516 DEGs upregulated and 499 downregulated in *GSTM1^-^* meningiomas (**Figure 2A**) and the primary tumors with *GSTM1* expression showed a trend towards clustering together (**Figure 2B**). Apart from expected differences in *GSTM1* expression, *GSTM1^-^* meningiomas showed downregulation of anterior gradient 2, protein disulphide isomerase family member ((*AGR2)* encoding a member of the disulfide isomerase family of endoplasmic reticulum proteins important for oxidative protein folding) (34), we also found upregulation of m*icrotubule associated protein 1 light chain 3 Gamma* ((*MAP1LC3C)* encoding a member of the microtubule-associated family of proteins that are essential in the formation of autophagosomes and lysosomal degradation of cargo), n*europeptide Y receptor Y6* (*NPY6R* regulator of the growth hormone axis and body composition) (35), and *NEDD4 E3 ubiquitin protein ligase* ((*NEDD4)* encoding an E3 ubiquitin ligase enzyme that targets proteins for ubiquitination) as shown in **Figure 2C**. It was observed that meningiomas in this cohort harbored either *GSMT1* or *MAP1LC3C* downregulation only and there were no samples with under expression of both (**Figure 3**). It has been shown that NEDD4 is frequently overexpressed in multiple human cancers and primarily functions as an oncogene in various malignancies (36), which aligns with its upregulation in recurrent meningiomas.

Gene set enrichment analyses revealed that pathways upregulated in *GSTM1^-^* meningiomas were related to cell-to-cell interaction and angiogenesis while downregulated pathways were related to transmembrane transport and intracellular signaling (**Figure 2D**; **Supplementary Figure S4**).

### 3.4 *GSTM1* null genotype in meningiomas with *GSTM1* downregulation

**Figure 4:**
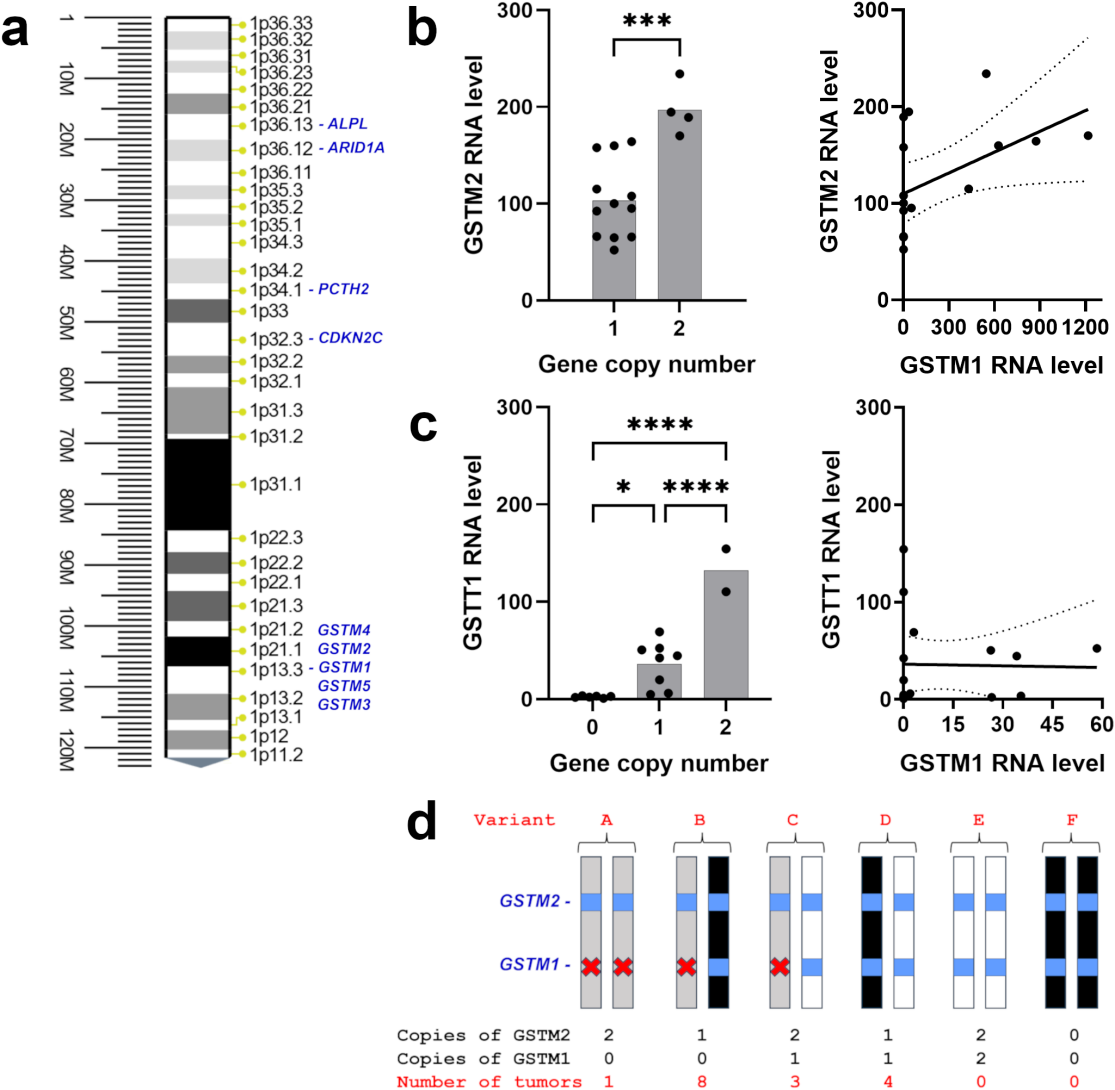
*GST(x)* gene expression and copy number in 1p^-^22q^-^NF2^-^ meningiomas. **(A)** Chr1p position of *GSTM1* and other related genes or genes of interest in meningioma. **(B)** (left) Scatter plot of *GSTM2* expression level and copy number. (right) Correlation of *GSTM2* expression with *GSTM1* expression plotted with linear regression line (solid) and 95% confidence interval (dotted lines). r=0.5021, p=0.0475. **(C)** (left) Scatter plot of *GSTT1* expression level and gene copy number. The y-axis displays relative quantity (RQ) multiplied by 1000. (right) Correlation of *GSTT1* expression with *GSTM1* expression plotted with linear regression line (solid) and 95% confidence interval (dotted lines). r=-0.0232, p=0.9321. **(D)** Schematic representation of *GSTM1* and *GSTM2* genotypes in 1p^-^22q^-^NF2^-^ meningiomas and the number of tumors in each group. White bars depict *GSTM1* present haplotype, black bars indicate somatic chr1p deletion in meningiomas, *GSTM1* and *GSTM2* genes are shown as blue squares, gray bars represent *GSTM1* null haplotypes, and deletion of *GSTM1* is indicated with a red x. *p<0.05; ***p<0.001; ****p<0.0001.

The expression of *GSTM1* on chromosome 1p13 (**Figure 4A**) is downregulated in recurrent 1p^-^22q^-^NF2^-^ meningiomas leading to a GSTM1 null genotype. To assess possible gene polymorphism as an explanation for differences in *GSTM1* expression, we evaluated *GSTM1* copy number variations (CNV) by multiplex quantitative polymerase chain reaction (qPCR). We also measured *GSTM2* CNV due to its proximity on chr1p and lack of reported polymorphism (37). All meningiomas with *GSTM1* under expression also showed copy number losses of *GSTM1* (**Table 2**). Four meningiomas were *GSTM2* homozygous at a copy number and none were recurrent tumors. *GSTM2* homozygous tumors also displayed a two-fold increase in *GSTM2* expression compared to tumors with one copy (**Figure 4B**, left), suggesting a proportional increase in expression to gene copy. Additionally, there was a significant positive correlation between *GSTM1* and *GSTM2* expression (Pearson r = 0.5021, 95% confidence interval = 0.0085 – 0.7999, p = 0.0475) (**Figure 4B**, right). This also aligns with the observation that *GSTM1^-^* meningiomas had significantly lower *GSTM2* expression versus *GSTM1^+^* meningiomas (**Supplementary File 1**).

**Table 2:** Relative expression* and copy numbers of *GST(x)* genes.

Tumor group	Patient ID	GSTM1		GSTM2		GSTT1		GSTT4	
		gene copy	mRNA level	gene copy	mRNA level	gene copy	mRNA level	gene copy	mRNA level
Recurrent	RF1	0	0.00	1	3.99	0	3.13	1	0.00
	RM1	0	0.00	1	5.70	1	4.75	1	0.00
	RM2	0	0.00	1	5.30	2	110.49	2	0.00
	RM4	0	0.00	1	4.27	0	1.98	1	0.00
Primary	PF1	1	35.46	2	15.21	0	3.71	1	0.00
	PF2	1	3.11	1	5.72	1	69.06	2	0.00
	PF3	0	0.00	1	2.73	0	2.79	1	0.00
	PF4	0	0.00	1	7.65	1	42.31	1	0.00
	PF5	1	26.37	1	7.05	1	50.56	1	0.00
	PF6	0	0.00	2	7.08	1	19.90	2	0.00
	PF7	0	0.00	1	3.95	2	154.33	2	0.00
	PM1	1	58.39	2	8.16	1	52.38	2	0.00
	PM2	1	1.97	2	10.89	1	6.03	2	0.00
	PM3	1	34.21	1	6.43	1	44.54	1	0.00
	PM4	1	26.62	1	6.80	0	2.23	2	0.00
	PM5	0	0.00	1	6.59	0	0.86	1	0.00

*GST gene mRNA levels were calculated relative to GAPDH mRNA levels measured by RNA-seq for GSTM genes and multiplex RT-qPCR for GSTT genes. Shown values represent the results multiplied by 1000.

Recurrent *GSTM1* null genotype meningiomas harbored either a) zero copies of *GSTM1* and two copies of *GSTM2*, indicating that *GSMT1* deletions in this patient were congenital (variant A), or b) were *GSTM2* hemizygous (variant B), suggesting that the loss of one *GSTM1* copy was inherited while another copy of the gene was lost in these tumors due to somatic deletion of chr1p (**Figure 4D**). Accordingly, recurrent meningiomas have inactivation of both copies of the gene either due to *GSTM1* or 1p13 deletions. *GSTM1* hemizygous non-recurrent meningiomas either had two *GSTM2* alleles or were hemizygous for *GSTM2*, where loss of a *GSTM1* copy was inherited (variant C) or lost due to somatic chr1p deletion (variant D). No meningiomas were homozygous for the wild type allele of *GSTM1* (variant E) or had both *GSTM1* and *GSTM2* somatic deletions (variant F).

### 3.5 Meningioma recurrence is independent of *GSTT1* expression

We also evaluated *GSTT1* polymorphism in our cohort as a gene mapping to cytogenetic band 22q11.23 which is near *NF2* (22q12.2) that is also frequently deleted in various cancers (31, 38). It is important to note that *GSTT1* is not included in the primary human genome assembly GRCCh38.p14 but is found in NT_187633 Chromosome 22 Reference GRCh38.p14 ALT_REF_LOCI_1 assembly. *GSTT1*, mapping to 22q11.23, copy number was heterogenous in recurrent tumors with two tumors having no *GSTT1*, one having one copy, and one having two copies (**Table 2**) as well as being heterogenous in non-recurrent meningiomas. We next examined CNV of *GSTT4*, which maps only 30 kilobases apart from *GSTT1* and does not display genetic polymorphism (37). Although none of 16 meningiomas displayed significant expression of *GSTT4*, nine tumors were *GSTT4* hemizygous, while the rest were homozygous (**Table 2**). Taken together with the fact that *GSTT4* is not usually deleted in humans, these results suggest that *GSTT4* hemizygous meningiomas lost a copy of the gene together with somatic deletion of chr22. Accordingly, the loss of another allele in tumors with *GSTT1* null genotype was inherited. *GSTT1* expression levels were measured by reverse transcription (RT) qPCR using *GAPDH* gene as an internal control. The analysis revealed that *GSTT1* expressed in a dose dependent manner, with the highest levels of expression in meningiomas with two copies of *GSTT1* (**Figure 4C**, left). Further, there was no correlation between *GSTM1* and *GSTT1* expression (Pearson r = -0.0232, 95% confidence interval = -0.5130 – 0.4780, p = 0.9321) (**Figure 4C**, right). Overall, *GSTT1* loss does not appear to be a biomarker of meningioma recurrence.

### 3.6 External validation of *GSTM1* as a biomarker of recurrent meningioma

With the availability of public datasets in the gene expression omnibus (GEO), we evaluated meningioma expression studies to determine if we could find evidence of *GSTM1* under expression in recurrent meningiomas. We identified five studies that clearly identified which tumors were from recurrent meningiomas. Lee et. al., 2010 (39), Clark et. al., 2013 (9), and Patel et. al., 2019 (13), each had expression data from 10-12 recurrent meningiomas and 58-145 primary tumors. Comparing expressions between the recurrent and primary tumors found no *GSTM1* under expression (Geo2R default analysis, adjusted p=0.233-0.970). In the remaining studies, we found significant differences in *GSTM1* expression between primary and recurrent tumors. For Schmidt et. al., 2016 (40), dataset, we were able to compare 31 recurrent and 23 primary tumors. Default Geo2R analysis found that there was a 98% decrease in *GSTM1* expression (adjusted p=0.00365) in the recurrent tumors. Similarly, when we analyzed the Cimino et. al., 2019 (41), dataset consisting of 5 recurrent and 3 primary tumors, we found a 3.4-fold decrease in *GSTM1* expression (adjusted p=0.00716) in the recurrent tumors. The main limitation of the public GEO datasets is that we could not determine which of the recurrent meningiomas were 1p^-^22q^-^NF2^-^. None the less, *GSTM1* under expression is found in recurrent meningiomas from independent datasets, externally validating *GSTM1^-^* as a biomarker for 1p^-^22q^-^NF2^-^meningioma recurrence.

## 4 Discussion

Meningiomas are the most common primary brain tumors, but we are currently limited in our ability to prevent recurrence or to identify tumors that may recur due to the lack of robust molecular biomarkers of recurrence. This work extends beyond classification and subclassification of primary meningiomas to characterize a distinct transcriptomic biomarker that is enriched in recurrent 1p^-^22q^-^NF2^-^ meningiomas. These meningiomas show the highest rate of recurrence using existing molecular classification but are still heterogenous with a substantial subset that do not recur. Our results demonstrate that 1p^-^22q^-^NF2^-^ meningiomas without expression of *GSTM1* comprise the recurrent 1p^-^22q^-^NF2^-^ meningiomas compared to those that express *GSTM1*, thereby addressing the heterogeneity in this existing molecular classification scheme.

These observations are in agreement with numerous previous studies, emphasizing *GSTM1* null genotype as a factor modulating the risk of developing cancer (42). For example, the *GSTM1* null genotype was associated with an increased risk of cervical cancer in Indian and Chinese populations (43). Moreover, a study comprising of 2500 individuals demonstrated that environmental risk factors significantly increase susceptibility to head and neck cancer in *GSTM1* null individuals (44). Importantly, colorectal cancer patients undergoing chemotherapy had higher survival rates even with only one copy of *GSTM1* (45). Taken together, these results imply that there is an association between cancer progression and *GSTM1* gene copy number, which is in general agreement with our observation that the expression of other genes from GST family (*GSTM2* and *GSTT1*) is also copy number dependent (**Figure 4**). Interestingly, there is a report that *GSTM2* expression may increase to compensate for the loss of *GSTM1* (46). However, we found no evidence for that mechanism in this study since *GSTM2* mRNA was positively correlated with *GSTM1* mRNA (**Figure 4B**, right, **Table 2**). Thus, loss of *GSTM1*’s ability to detoxify electrophiles generated by xenobiotic-induced reactive oxygen species, along with the lack of compensation by other GST genes, increases cancer susceptibility and prompts further malignization of tumors, including meningiomas.

The association of *GSTM1* and *GSTT1* genetic polymorphism with the development of meningiomas has been previously examined. Whereas the *GSTT1* null genotype was significantly associated with the risk of meningioma, no significant effect of *GSTM1* genotypes on susceptibility was identified (47, 48). These results contradict our current findings of all recurrent meningiomas in our cohort displaying a *GSTM1* null genotype, with no difference in *GSTT1* genotypes in our study. Discrepancy with our results may be explained by our study focusing on the differences between recurrent and primary 1p^-^22q^-^NF2^-^ meningiomas. Another difference between the studies was that the majority of meningiomas in our group with *GSTM1* null genotype had only one copy of the gene lost due to inherited deletion of the gene, while another copy may have been lost due to somatic chr1p deletion. Whether a somatic chr1p deletion contained present or null allele remains to be determined by further investigations, which will require the analysis of germline *GSTM1* copy numbers and should include a larger cohort of patients. Future experiments are needed to examine the existence of a significant relationship between *GSTM1* copy numbers and meningioma development.

The comparison of recurrent and primary meningiomas transcriptomes in a gene-set enrichment analysis revealed that recurrent meningiomas were characterized by overexpression of gene sets involved in cell structure and signaling and under expression of developmental pathways. Additionally, a gene-set enrichment analysis of *GSTM1* null meningiomas versus *GSTM1* expressing meningiomas identified cell-to-cell interaction and angiogenesis pathways upregulated as well as transmembrane transport and intracellular signaling pathways downregulated in *GSTM1^-^* meningiomas. The most significant DEG in *GSTM1* null meningiomas was upregulation of *MAP1LC3C* (**Figure 2**), which is located on chr1q43, and is required for the formation of autophagosomal membranes for autophagy-related processes and cell homeostasis (49), but is not routinely deleted in meningiomas (18). *MAP1LC3C* is present in all vertebrates except rodents, making the generation of *MAP1LC3C*-deficient mice impossible. *MAP1LC3C* has been shown to play a tumor-suppressing role in breast cancer (50) and renal clear cell carcinoma (51) development. Future experiments may offer a better understanding of the molecular mechanism of MAP1LC3C function in tumor cells and test whether it plays a positive role in meningioma development.

Another DEG upregulated in *GSTM1^-^* meningiomas compared to *GSTM1^+^* tumors is *NEDD4* (**Figure 2**), which encodes a member of HECT family of E3 ubiquitin ligases. NEDD4 is an evolutionarily conserved protein highly expressed in the early embryonic brain (52) where it promotes the development of neural dendrites (53), and results in neonatal lethality with targeted deletion (54), suggesting that NEDD4 is involved in neurodevelopment including meninges growth and organization. Additionally, NEDD4 is a proto-oncogene that downregulates *PTEN* expression, thereby impacting the PI3K/AKT/mTOR pathway that is implicated in multiple types of cancers (55). It will be important for future studies to further evaluate these additional pathways and DEGs to identify potential additional biomarkers of meningioma progression beyond *GSTM1* as well as potential novel therapeutic targets.

In addition to detoxification, *GSTM1* has been shown to modulate inflammation though NF-kB, GM-CSF (granulocyte–macrophage colony stimulating factor) and CCL2 (chemokine [C–C motif] ligands 2) signaling (56, 57). *GSTM1* can also activate STAT3 signaling, a key modulator of multiple cellular processes including proliferation, apoptosis, and metastasis, which is altered in multiple types of cancers (56, 58, 59). The family of GSTs have also been demonstrated to affect signal transduction mechanisms involved in cell proliferation and apoptosis though modulation of c-Jun-N-terminal kinases (JNKs) and apoptosis signal-regulating kinase (ASK1) (57). Loss of *NF2* leads to disruption of Merlin, a key tumor suppressor that targets multiple signaling pathways such as mTORC1 (cell growth and metabolism in response to environmental factors), Ras/Rac/PAK (cellular proliferation, differentiation, and transformation), and CRL4-DCAF (DNA damage response and mitotic exit) (60, 61). Thus, we can begin to build synergistic models between *GSTM1* and *NF2* signaling. Loss of *GSTM1* leads to accumulation of intracellular toxins, which could be enhanced through dysregulated mTOC1 signaling. The toxins could then lead to DNA damage, worsened by disruption of CRL4, which allows the cell to escape apoptosis via STAT3, JNKs, and/or ASK1 signaling. Further, proliferation signals from STAT3 and the Ras/Rac/PAK pathways would be pro-oncogenic. While this model is partially supported by the increases in *MAP1LC3C* and *NEDD4* expression in the *GSTM1^-^* meningiomas, future studies will be needed to investigate the various components of this model at both the mRNA and protein level.

The results of our study extend beyond understanding tumor development for the novel purpose of identifying a biomarker of tumor recurrence, which is crucial for informing clinical treatment decisions. A biomarker of meningioma recurrence has the potential to shape personalized medicine approaches in the future by allowing clinicians to match the extent of initial meningioma treatment to the risk for later recurrence based on the presence or absence of a *GSTM1* null genotype. One postulated explanation for the relationship between *GSTM1* under expression and meningioma recurrence is that *GSTM1* function is necessary to remove substances secreted by meningiomas that stimulate their growth and without this host factor present meningioma growth is relatively less controlled. This work is limited to transcriptomic analysis and further validation is required at the protein expression level. Another likely limitation of this pilot study is that the examination of meningioma recurrence was limited to 1p^-^22q^-^NF2^-^ tumors and did not include analysis of other meningioma subtypes, including 14q^-^22q^-^ group tumors. Since measuring *GSTM1* expression in additional tumor subtypes will allow us to determine if it is a common biomarker for recurrent meningiomas, we plan to analyze 14q^-^22q^-^ and other subtypes of meningiomas in our future follow-up studies. Follow-up studies are required to evaluate *GSTM1* as a molecular biomarker of recurrence prospectively and to assess its impact on treatment decisions and patient outcomes.

## Data Availability

The original contributions presented in the study are publicly available. This data can be found here: GSE283616. https://www.ncbi.nlm.nih.gov/geo/query/acc.cgi?acc=GSE283616.

## Supplementary material

Supplementary Figure 1 Hierarchical clustering of DEGs in groups between recurrent (orange planks) and primary (pink planks) meningioma samples. Columns corresponding to each meningioma are indicated at the bottom. Green and red colors indicate low and high relative mRNA expression levels, respectively.

Supplementary Figure 2 Heat map of Pearson correlation between all tested meningioma samples. The change of the square (R^2^) value of the correlation coefficient of Pearson is indicated by the change of the blue color. Each grid in the figure represents the correlation between two samples; different colors represent correlation between samples. The deeper color indicates a bigger R^2^ value and a higher correlation between samples.

Supplementary Figure 3 Bubble plot of Reactome **(A)** and KEGG **(B)** enrichment analysis of signaling pathways upregulated in recurrent (left panel) or primary (right panel) meningiomas. Each bubble represents a pathway. Gene ratio (x-axis) is the percentage of significant genes over the total genes in each pathway.

Supplementary Figure 4 Bubble plot of Reactome **(A)** and KEGG **(B)** enrichment analysis of signaling pathways upregulated in recurrent (left panel) or primary (right panel) *GSTM1^-^* meningiomas. Each bubble represents a pathway. Gene ratio (x-axis) is the percentage of significant genes over the total genes in each pathway.

Supplementary File 1 Recurrent meningioma *vs*. Primary meningioma differently expressed genes (DEG).

Supplementary File 2
*GSTM^+^* meningioma *vs*. *GSTM^-^* meningioma differently expressed genes (DEG).
