# Supplementary figures and images for: GSTM1 null genotype underpins recurrence of NF2 meningiomas
Source: Front Oncol. 2024 Dec 12;14:1506708. doi: 10.3389/fonc.2024.1506708 (PMC11669715; doi:10.3389/fonc.2024.1506708)

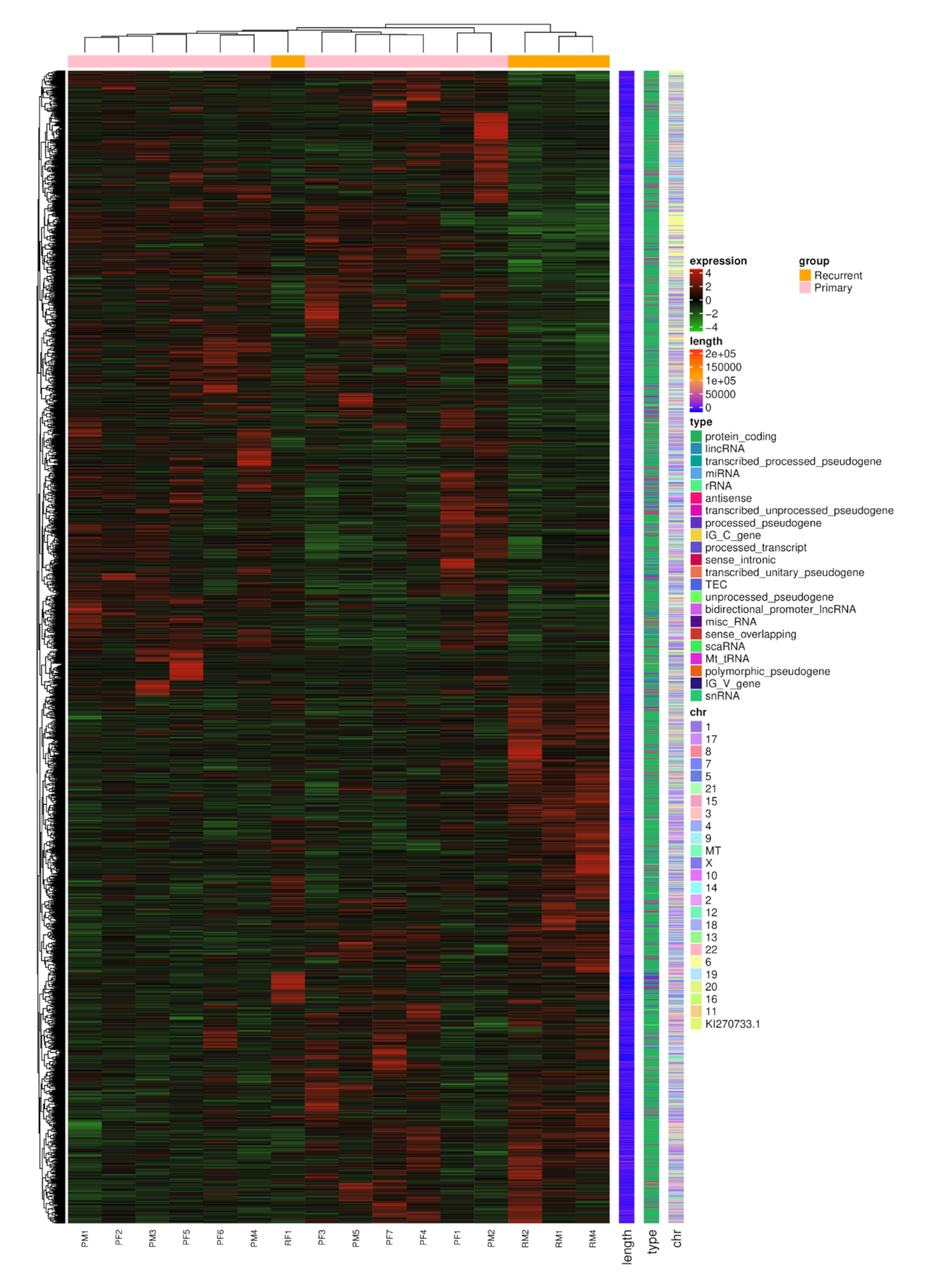

Supplement: Supplementary Figure 1 — Hierarchical clustering of DEGs in groups between recurrent (orange planks) and primary (pink planks) meningioma samples. Columns corresponding to each meningioma are indicated at the bottom. Green and red colors indicate low and high relative mRNA expression levels, respectively. [file Image1.tif]

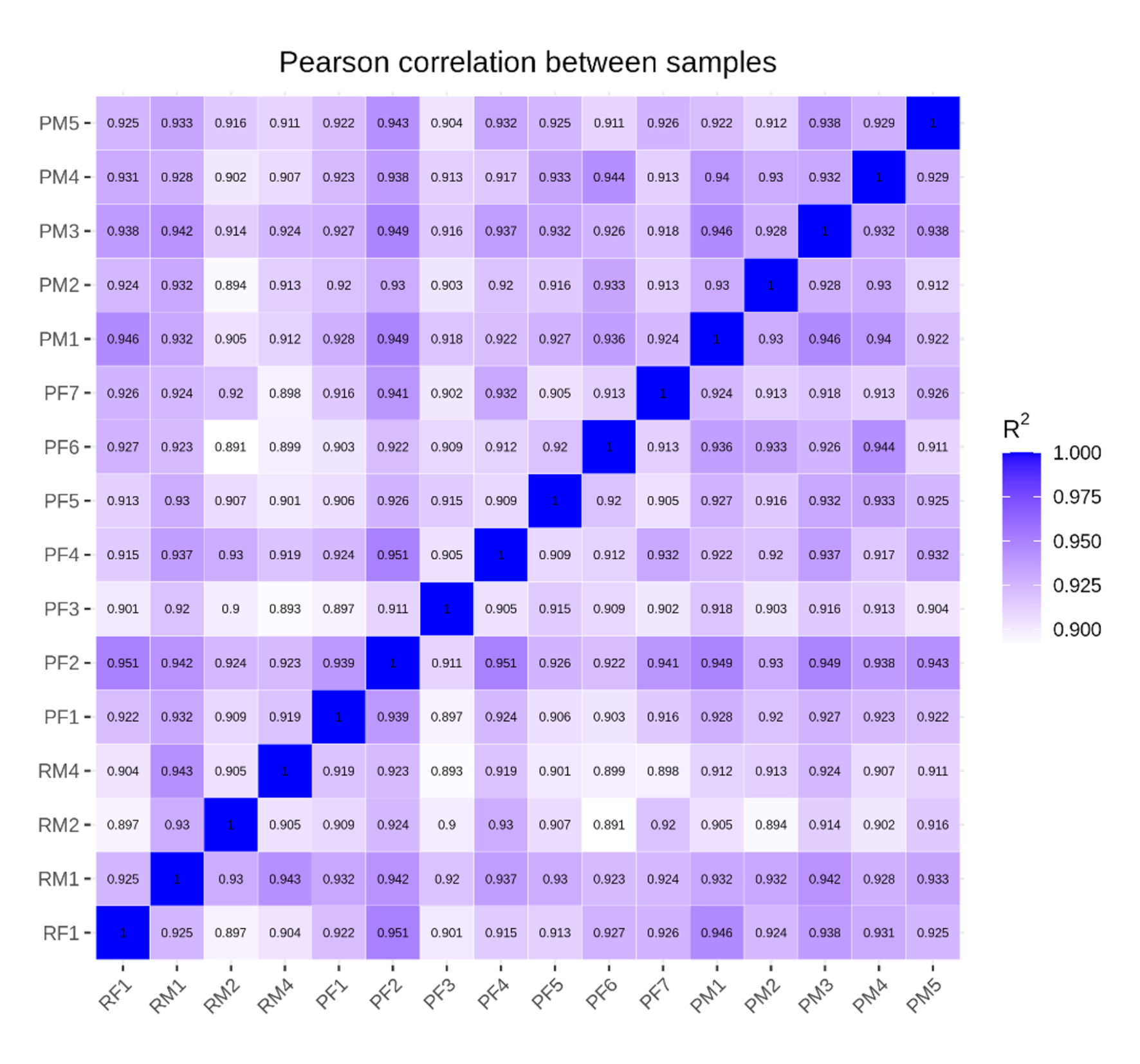

Supplement: Supplementary Figure 2 — Heat map of Pearson correlation between all tested meningioma samples. The change of the square (R2) value of the correlation coefficient of Pearson is indicated by the change of the blue color. Each grid in the figure represents the correlation between two samples; different colors represent correlation between samples. The deeper color indicates a bigger R2 value and a higher correlation between samples. [file Image2.tif]

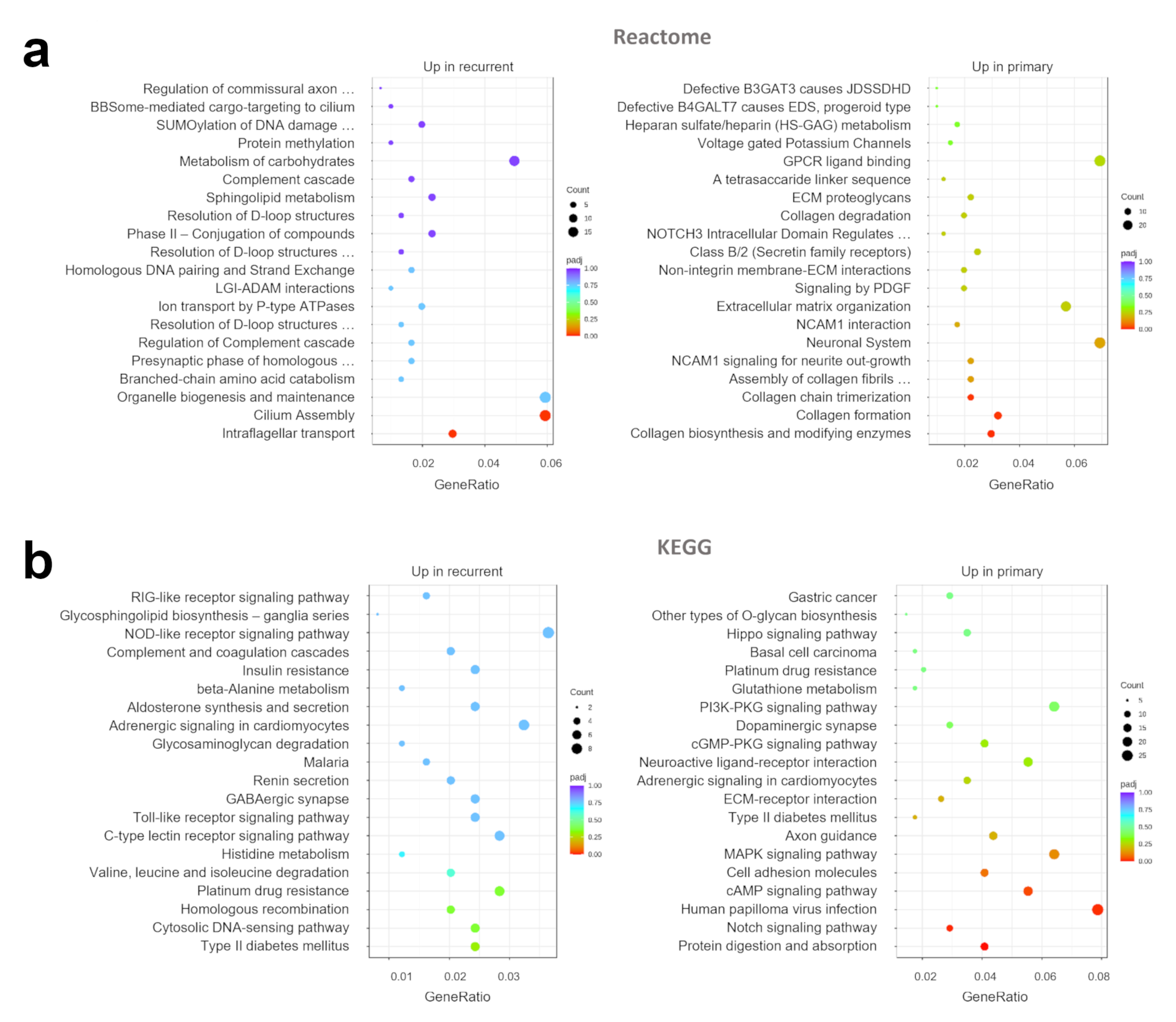

Supplement: Supplementary Figure 3 — Bubble plot of Reactome (A) and KEGG (B) enrichment analysis of signaling pathways upregulated in recurrent (left panel) or primary (right panel) meningiomas. Each bubble represents a pathway. Gene ratio (x-axis) is the percentage of significant genes over the total genes in each pathway. [file Image3.tif]

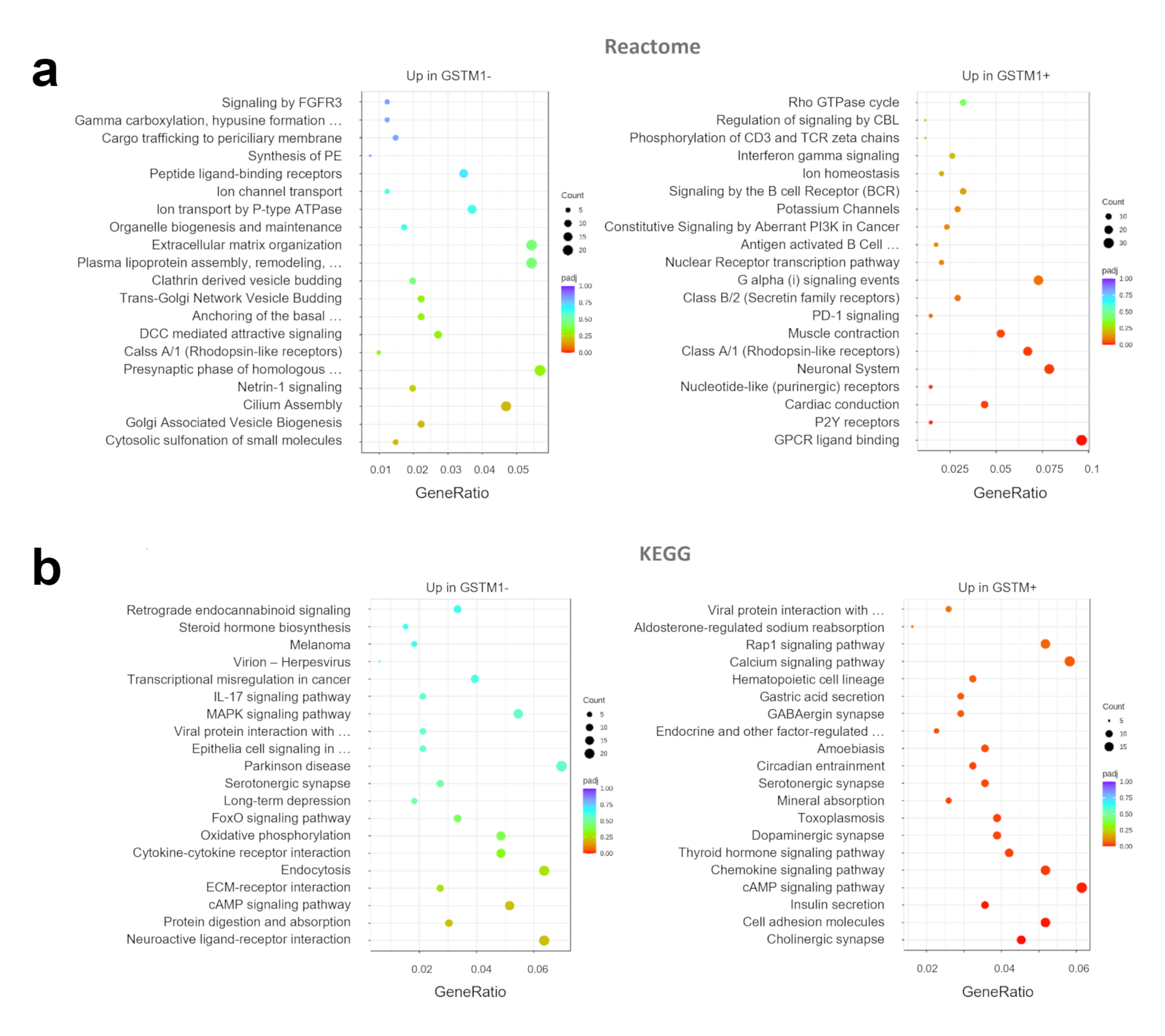

Supplement: Supplementary Figure 4 — Bubble plot of Reactome (A) and KEGG (B) enrichment analysis of signaling pathways upregulated in recurrent (left panel) or primary (right panel) GSTM1- meningiomas. Each bubble represents a pathway. Gene ratio (x-axis) is the percentage of significant genes over the total genes in each pathway. [file Image4.tif]
